# Possible involvement of integrin-mediated signalling in oocyte activation: evidence that a cyclic RGD-containing peptide can stimulate protein kinase C and cortical granule exocytosis in mouse oocytes

**DOI:** 10.1186/1477-7827-4-48

**Published:** 2006-09-25

**Authors:** Carla Tatone, Maria Cristina Carbone

**Affiliations:** 1 Department of Biomedical Sciences and Technologies, University of L'Aquila, Via Vetoio, 67100 L'Aquila, Italy

## Abstract

**Background:**

Mammalian sperm-oocyte interaction at fertilization involves several combined interactions between integrins on the oocyte and integrin ligands (disintegrins) on the sperm. Recent research has indicated the ability of peptides containing the RGD sequence that characterized several sperm disintegrins, to induce intracellular Ca2+ transients and to initiate parthenogenetic development in amphibian and bovine oocytes. In the present study, we investigate the hypothesis that an integrin-associated signalling may participate in oocyte activation signalling by determining the ability of a cyclic RGD-containing peptide to stimulate the activation of protein kinase C (PKC) and the exocytosis of cortical granules in mouse oocytes.

**Methods:**

An In-Vitro-Fertilization assay (IVF) was carried in order to test the condition under which a peptide containing the RGD sequence, cyclo(Arg-Gly-Asp-D-Phe-Val), was able to inhibit sperm fusion with zona-free mouse oocytes at metaphase II stage. PKC activity was determined by means of an assay based on the ability of cell lysates to phosphorylate MARKS peptide, a specific PKC substrate. Loss of cortical granules was evaluated by measuring density in the oocyte cortex of cortical granules stained with LCA-biotin/Texas red-streptavidin. In all the experiments, effects of a control peptide containing a non RGD sequence, cyclo(Arg-Ala-Asp-D-Phe-Val), were evaluated.

**Results:**

The IVF assay revealed that the fusion rate declined significantly when insemination was carried out in the presence of cyclic RGD peptide at concentrations > or = 250 microM (P < 0.05, Student-Newman-Keuls Method). When the peptide was applied to the oocytes at these concentrations, a dose-dependent increase of PKC activity was observed, in association with a loss of cortical granules ranging from 38+/-2.5 % to 52+/-5.4 %. Evaluation of meiotic status revealed that cyclic RGD peptide was ineffective in inducing meiosis resumption under conditions used in the present study.

**Conclusion:**

The presents results provide evidence that a cyclic RGD peptide highly effective in inhibiting sperm-oocyte interaction stimulates in mouse oocytes the activation of PKC and the exocytosis of cortical granules. These data support the view that RGD-binding receptors may function as signalling receptors giving rise integrated signalling not sufficient for a full oocyte activation response. This study may contribute to the understanding of possible negative effects of skipping gamete interaction in IVF techniques.

## Background

At fertilisation the oocyte undergoes a series of rapid changes responsible for the onset of the embryonic development and the blockage of polyspermy. These changes, collectively known as "oocyte activation", are under the regulation of cytoplasmic signalling events activated in the oocyte following a multi-step interaction with the fertilising sperm [1-3]. It is well established that upon fusion sperm releases into the oocyte a sperm-specific phospholipase C-zeta (PLCζ) which induces a rise in intracellular Ca^2+ ^capable of releasing metaphase arrest and driving the zygote through the embryonic cell cycle [4]. Although the Ca^2+^-mediated signal transduction pathway at fertilization is not fully resolved, it seems to involve specific kinases such as protein kinase C (PKC) known to be activated in many cell types through enzyme- or G-protein-coupled receptors localised on plasma membrane [5,6]. However, due to the effectiveness of intracytoplasmic sperm injection in most mammalian species [7], the hypothesis that receptor-mediated pathways may participate in the oocyte activation process has been poorly investigated.

It is well established that binding of sperm ligands to specific oolemma receptors is a prerequisite step in sperm-oocyte interaction leading to fertilisation [8,9]. Candidate molecules involved in gamete interactions include integrins, transmembrane glycoproteins with heterodimeric structure (alpha-chain and beta-chain) that act as co-receptors in many cell-cell interaction [10]. Individual integrins can bind to more than one ligand and about half of them recognize the tripeptide sequence Arg-Gly-Asp (RGD) present in the extracellular matrix proteins such as fibronectin and vitronectin [11]. Integrins expressed on the surface of mouse oocytes can be divided into two groups: β 1 integrins (α2 β1, α3β1, α5 β1, α6 β1 and α9β1) and αv integrins (αv β1, αvβ3, αv β5; [12,13]). Integrin recognition sequences known to play a role in fertilization are the RGD sequence and other tripeptide sequences such as TDE, QDE and FEE included in the active site of fertilin beta, a component of the first molecule identified as a sperm surface protein required for sperm-oocyte fusion [14-18]. Recently it has been suggested the hypothesis that sperm-oocyte binding and fusion involve combined interactions between RGD-sensitive integrins such as αv β1 and RGD-insensitive integrins such as α6 β1 integrins on the oocyte [19].

In order to clarify the role of integrins at fertilization, it is important to consider that these molecules can serve not only as structural receptors that participate in cell-cell and cell-matrix interaction, but also as signalling receptors that regulate intracellular pH [20], intracellular free Ca^2+ ^[21], inositol lipid turnover [22] and protein phosphorylation [23]. Recent research has indicated the ability of peptides containing a RGD sequence to induce intracellular Ca^2+ ^transients and to initiate parthenogenetic development in amphibian and bovine oocytes [24-26], indicating that RGD-binding receptors may function as signalling receptors in oocytes as it occurs in other cell types. Multiple intracellular signalling molecules are stimulated following integrin-dependent adhesion. These include members of mitogen-activated protein kinase (MAPK) signalling pathways, Rho family GTPases, non-receptor tyrosine kinases such as focal adhesion kinase (FAK) and Src, and members of the lipid signalling pathways such as phosphatidyl-inositol 3-kinase (PI 3-K), and protein kinase C [27-29]. PKC signalling is considered a major regulator of oocyte activation acting both dependently and independently from the fertilization calcium signal [30,31]. Although its role is not clearly established, it has been proposed that this kinase provides integrated signals aimed to modulate the kinetics and the extent of activation events such as Ca^2+ ^spiking and cortical granule exocytosis [32].

Based on the above observations, we put forward the hypothesis that integrins may participate in the activation-associated signalling in mouse oocytes. To this end, in the present study we investigated the ability of a cyclic RGD peptide to activate a pathway leading to the stimulation of protein kinase C and cortical granule exocytosis.

## Methods

### Reagents

All reagents were purchased from Sigma Chemical Company (St. Louis, MO) unless otherwise stated.

### Peptides

The peptide containing the RGD sequence cyclo(Arg-Gly-Asp-D-Phe-Val) [33] and the control peptide containing a non RGD sequence cyclo(Arg-Ala-Asp-D-Phe-Val) [34], were purchased from Peptide International, Inc. (Louisville, Kentucky). As demonstrated by the manufacturer when tested by thin layer chromatography both the peptides showed a single spot. Lyophilised peptides were resuspended in T6 medium [35] at 500 μM concentration as indicated in the datasheet, aliquotted and stored at -20°C and used within 3 weeks.

### Oocyte and sperm isolation

Random bred Swiss CD1 female mice (22–25 days old, Charles River, Como, Italy) were superovulated by intraperitoneal injection of 7.5 I.U. PMSG and 7.5 I.U. hCG 48 hr apart. After 14 hr mice were killed by cervical dislocation. Oocytes were released from oviducts in M2 medium [35] and the cumulus cells were dispersed by a brief exposure to 0.3 mg/ml hyaluronidase. The zonae pellucidae were removed by treatment (approximately 1 min) with Tyrode's solution [36] and the zona-free oocytes were cultured at 37°C, 5% CO_2 _in M16 medium for 1 hr before their use in the experimental groups.

Spermatozoa were obtained by excising the caudae epididymides from two adult CD1 males (3–5 months old, Charles River, Como, Italy) as previously described [31].

### In vitro fertilisation and sperm fusion assay

The sperm suspension was diluted to obtain 50 μl insemination drops containing 1–5 × 10^4 ^sperm/ml covered with mineral oil. About 25 oocytes preincubated for 30 min in T6 containing peptides at different concentrations or in T6 alone were incubated in each drop and maintained in the incubator at 37°C in 5% CO_2 _for 10–30 min. Inseminated oocytes were freed from loosely associated sperm before processing for the evaluation of the designated parameters. Further development of fertilised oocytes was carried out in 50 μl drops of M16 medium.

To visualise sperm-oocyte fusion after fertilisation we used the dye transfer technique as previously described [37]. In this assay, upon fusion with oocytes preloaded with the DNA-staining dye Hoechst 33342, sperm nuclei become brightly fluorescent as the dye gains access and binds sperm DNA. Oocytes were loaded with Hoechst 33342 (0.1 μg/ml) by a 15 min incubation, rinsed thoroughly in T6 medium and immediately incubated with sperm. After 10 min of insemination, oocytes were collected and washed in M2 before being fixed by a 15 min incubation in 3.7% paraformaldehyde in PBS. Fixed oocytes were mounted on slides and scored for the presence of fused sperm under a microscope fitted for epifluorescence (Leitz Dialux, Leitz, Wien, Austria).

### Protein kinase C assay

PKC activity was assayed using the protocol of Gallicano et al. [30]. For each reaction, groups of 10 oocytes, collected at different times of insemination, were washed in collection buffer (phosphate buffered saline (PBS) containing 1 mg/ml polyvinyl alcohol, 5 mM EDTA, 10 mM Na_3_VO_4_, 10 mM NaF), transferred to a centrifuge tube in 2 μl of collection buffer and immediately submerged in liquid N_2 _to flash-freeze the oocytes, followed by storage at -80°C until the kinase assay was performed. The frozen oocytes were thawed in 10 μl PKC buffer which contained β-glycerophosphate (54 mM), para-nitrophenylphosphate (14.5 mM), MOPS (24 mM), MgCl_2 _(14.5 mM), EGTA (14.5 mM), EDTA (0.12 mM), DTT (1 mM), leupeptin (1μg/ml), aprotinin (1 μg/ml), ML-9 (10 μM), genestein (75 μM), chimostatin (1 μg/ml), tripsin-chimotripsin inhibitor (1 μg/ml), PKI (2.4 μM), 50 μCi/ml ν-[^32^P]ATP(Amersham Pharmacia Biotech, Italy) and MARKS (2.5 mg/ml, BIOMOL, Plymouth Meeting, PA) as a specific substrate. After 30 min at 37°C, assays were stopped by adding tricine sample buffer (1:1) (Bio-Rad Laboratories, Hercules, CA). Samples were electrophoresed and the gel was subjected to autoradiography as previously described [38]. To inhibit PKC activity, BIM was added to the assay buffer at a final concentration of 10 μM (control). Enzyme activity was semiquantified densitometrically using a Bio-rad GS-670 computerised imaging densitometer and Molecular Analyst software (Bio-rad Laboratories, Hercules, CA). Intensity of bands were quantified after background subtractions. For each autoradiogram, 3 replicates were performed with 10 oocytes per time-point per replicate and ratios of mean band density in the experimental groups to that of BIM treated samples (control) were evaluated. PKC activity at different time points was expressed as mean ± SEM of ratios obtained from at least 3 autoradiograms.

### Staining and quantification of cortical granules

Oocytes were fixed in a 3.7% (w/v) paraformaldeyde and, after permeabilization with Triton X-100, were incubated with LCA (Lens Culinaris Agglutinin)-coupled to biotin and then with Texas red-streptavidin as previously described [38]. Briefly, the oocytes were mounted on slide in 50% w/v glycerol and CGs in the cortex were visualised by a fluorescence microscope equipped with a 100x objective and oil immersion. The CG density for each oocyte was computed by image analysis based on the same principles as manual counting described previously. The images on flat optical fields of cortex resulting from partial compression of the oocyte by the coverslip, were captured by a Vario Cam monochrome CCD and then transferred to a PC with image analysis software (KS300, Kontron Elektronik Gmbh, Germany) [38]. The density of CGs per 100 μm^2 ^for each oocyte was computed by image analysis as the mean of the counts from tree equal areas of cortex containing cortical granules. For each group, the percentage loss of CGs from the cortex was calculated by comparing the mean density of CGs of the treated group with the mean density of CGs of the untreated control group, according to the following equation: %CG loss = 1-[density of CGs in treated group/density of CGs in untreated group] × 100.

### Evaluation of oocyte activation

Oocytes fixed in paraformaldehyde were stained with 3 mg/ml Hoechst 33342 for 10 min, mounted on slides and monitored under an epifluorecence microscope for the presence of chromosomes at anaphase and telophase or pronuclei. As a positive control for oocyte activation, oocytes were activated with 7% ethanol [37].

### Statistical analysis

Each group of experiments was repeated at least three times and data are presented as mean ± SEM, unless stated otherwise. Multiple comparison of values were analysed using Student-Newman-Keul's test (SigmaStat software; Jandel Scientific Software Corporation, San Rafael, CA). Proportions were compared using z-test. Differences associated with a P value lower than 0.05 were considered statistically significant.

## Results

### Effect of a cyclic RGD containing peptide on sperm-oocyte interaction

**Figure 1:**
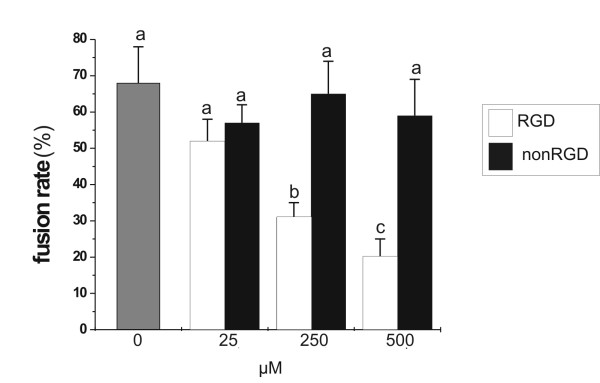
**Effects of a cyclic RGD peptide on sperm-oocyte fusion**. ZP-free mouse oocyte were incubated in the presence of different concentrations of cyclic RGD or nonRGD peptides for 30 min prior to fertilization. Oocytes were then inseminated with 1–5 × 10^4 ^sperm/ml in the presence of peptide. The results here represent the average of 3 experiments per peptide concentration ± S.E.M. and a total of 60–80 inseminated oocytes per experimental point. Means with different letters are statistically different (P < 0.05).

As in the mouse RGD containing peptides are known to interfere with sperm-oocyte interaction [16,17], we tested the conditions under which a cyclic RGD peptide interacts effectively with the oocyte by monitoring its ability to inhibit fertilization. To this end we performed an IVF assay where successful gamete interaction was assessed by monitoring sperm fusion. As shown in Figure 1, the IVF assay revealed that fusion rate declined significantly when insemination was carried out in the presence of cyclic RGD peptide at 250 μM (P < 0.05). A further increase to 500 μM was responsible for a further reduction of fusion rate to about 20%, a value 3-fold lower than that observed in the presence of the same concentration of the control peptide. Since the control cyclic peptide failed to affect fertilisation at any concentration tested (Figure 1), present results were taken an indirect evidence of the peptide interaction with integrin receptors on mouse oocytes.

### PKC activity in mouse oocytes exposed to a cyclic RGD containing peptide

**Figure 2:**
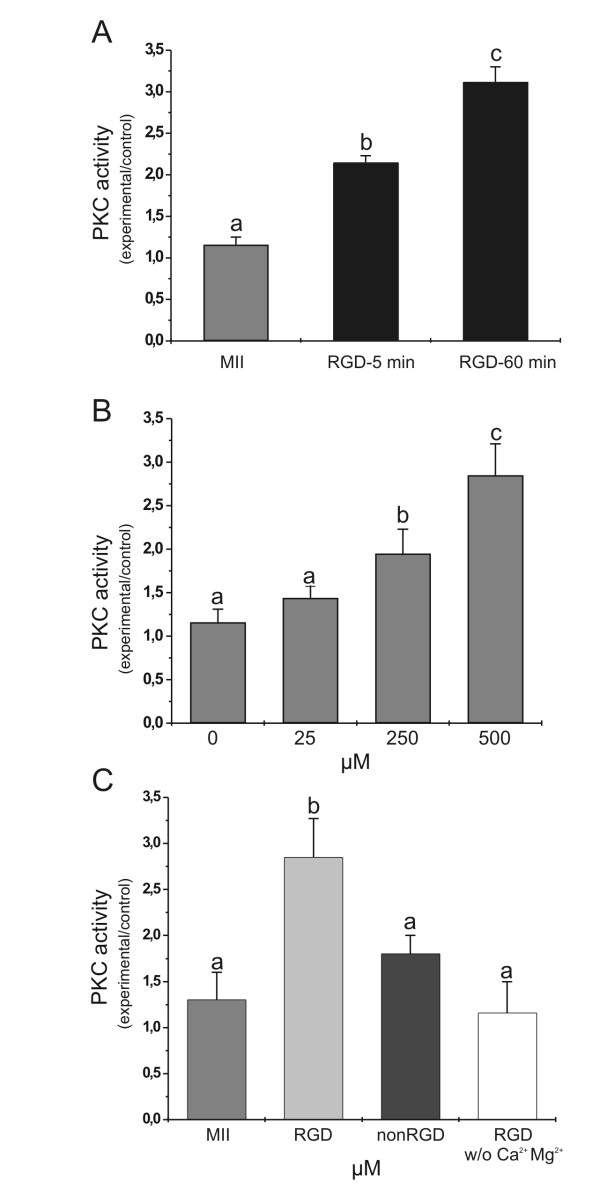
**Effects of a cyclic RGD peptide on PKC activity**. PKC activity in ZP-free mouse oocytes incubated in the presence of cyclic RGD or nonRGD peptides. A, oocytes incubated in the presence of 500 μM RGD for 5 min or 60 min and processed for PKC assay. B, oocytes incubated in the presence of different concentrations of cyclic RGD peptide for 60 min. C, oocytes exposed for 60 min to 500 μM RGD peptide in the presence or in the absence of Ca^2+ ^and Mg^2+^, and to 500 μM nonRGD peptide. PKC activity is expressed as mean ± SEM of ratios of density in the experimental group to that of BIM treated samples. Means with different letters are statistically different (P < 0.05).

In further experiments we applied the peptide to the oocytes at the concentration of 500 μM and then subjected them to a PKC assay. Results from these assays showed that the peptide exposure resulted in a significant stimulation of the enzyme. This effect was seen as early as 5 min post-treatment, and after 60 min the PKC activity reached a level about three-fold higher than that observed in untreated oocytes (time 0; Figure 2A). As shown in Figure 2B, a significant increase in PKC activity could be observed when RGD concentration was lowered to 250 μM although the level of activity was reduced as compared with that observed at 500 μM. When oocytes were exposed to the non RGD-peptide PKC activity did not significantly increase as compared with that monitored in untreated MII oocytes (Figure 2C). As shown in the same figure, no change was seen when the treatment with the cyclic RGD peptide was carried out in a Mg^2+ ^and Ca^2+^-free medium, a condition that prevent ligand-integrin interaction [17,39].

### Cortical granule exocytosis and meiotic status in mouse oocytes exposed to a cyclic RGD containing peptide

**Figure 3:**
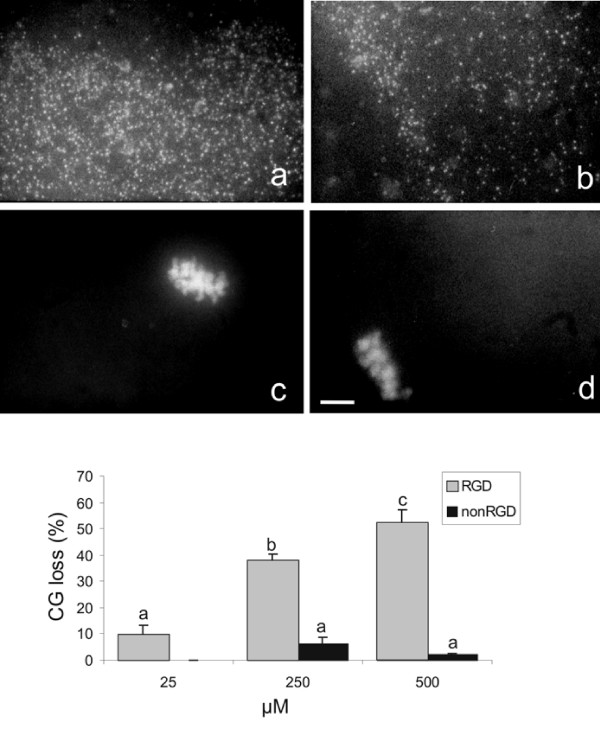
**Cortical granule exocytosis in oocytes exposed to a cyclic RGD peptide**. ZP-free mouse oocytes were incubated in the presence of different concentrations of cyclic RGD or nonRGD peptides for 60 min and stained with LCA-biotin/Texas red-streptavidin 2 hr later. A) Representative photomicrographs of cortical granules localized in the cortex and corresponding chromosomes of a MII oocyte (a, c) and an oocyte exposed to the RGD peptide (b, d). Final magnification, ×1250. B) Histogram showing CG loss in oocytes exposed to cyclic RGD or nonRGD peptides. The results here represent the average of 3 experiments per peptide concentration ± S.E.M. and a total of 60–80 inseminated oocytes per experimental point. Bar = 10 μm. Means with different letters are statistically different (P < 0.05).

To establish whether the cyclic RGD peptide could induce cortical granule exocytosis, mouse oocytes were incubated in the presence of different concentrations of cyclic RGD- and nonRGD peptides for 1 hr and processed for the evaluation of loss of cortical granules 2 hr later. As shown Figure 3, following the exposure to the cyclic RGD peptide, the oocytes underwent CG exocytosis in a dose-dependent manner. The loss of cortical granules ranged from 38 ± 2.5 % at 250 μM to 52 ± 5.4 % at 500 μM. Both these value were significantly higher than that monitored in oocytes exposed to the nonRGD peptide. As shown in the representative micrographs in Figure 3b and 3d, evaluation of meiotic status revealed that cyclic RGD peptide was ineffective in inducing meiosis resumption under conditions used in the present study. To confirm this, in further experiments we monitored the presence of oocytes at anaphase, telophase or pronuclei stage (activated oocytes) following 8 hr from the exposure to 500 μM cyclic RGD- or nonRGD peptides and to 7% ethanol, as a positive control. As shown in Table 1, the percentage of oocytes treated with the cyclic RGD peptide and showing meiosis resumption was statistically no different from than those exposed to the nonRGD peptide or untreated.

**Table 1:** Analysis of mouse oocyte activation at 8 hr after the exposure to a cyclic RGD peptide

Treatment	No. oocytes	No. activated (%)
no peptide	95	8 (8%)^a^
7% ethanol	105	86 (82%)^b^
cyclic RGD 500 μM	140	10 (7%)^a^
cyclic nonRGD 500 μM	110	9 (8%)^a^

^a, b ^Different letters indicates values statistically different (P < 0.001).

## Discussion

In this study, we provide evidence that exposure of mouse oocytes to a cyclic RGD peptide can inhibit fertilization and induce an activation-like response which includes the activation of PKC signalling and exocytosis of cortical granules. To our knowledge, this is the first report identifying a potential role of integrins and their ligands in the signalling events underlying mouse oocyte activation.

It is well known that in the mouse RGD-containing peptides do not have a substantial inhibitory effect on sperm-oocyte interaction as it occurs in other species, but cause a partial inhibition of fertilization, an observation taken as an evidence that sperm-oocyte fusion would utilize multiple molecules and/or multiple sites on molecules [16,17]. Although in our IVF assay a complete inhibition of sperm fusion was not achieved, the cyclic RGD peptide we employed reveals a high biological activity being about 50% inhibition observed at 250 μM, a concentration ten-fold lower than that required with linear RGD peptides[17]. Nevertheless, we are not able to establish whether higher concentrations of the peptide would have been more effective since in this study the lyophilized compound was dissolved at the maximal concentration allowed according to manufacturer's instructions. However, given that the RGD peptide inhibited sperm interaction whereas the nonRGD did not, we have taken these results as an indirect evidence of peptide binding to integrin receptors.

In studies of integrin functions in gametes and somatic cells, synthetic peptides containing the RGD (Arg-Gly-Asp) motif have been extensively used as the inhibitors of integrin-ligand interactions. Although the inhibitory activity of disintegrins depends mainly from their primary structure, structural and functional studies suggest that the receptor binding ability of these proteins lies in subtle positional requirements of the tripeptide RGD that is harboured in a defined hairpin loop (the disintegrin loop) projecting from the disintegrin core. This has led to the study of small, chemically synthesised, cyclic-RGD peptides, which exert more potency than linear RGD in integrin binding assay [40]. Thus it is likely that the cyclic RGD peptide used in the present study mimics the physiological action of RGD-containing proteins, supporting the view that, along with proteins with other tripeptide sequence such as fertilin [14], RGD-containing polypeptides located on sperm membrane, such as vitronectin [41,42], play an important role in gamete interactions leading to fertilization.

The analysis of PKC activity in oocytes exposed to the cyclic RGD peptide at concentrations effective in inhibiting sperm fusion revealed a significant increase in the activity of this enzyme. This finding supports the hypothesis that under this condition an oocyte-integrin signalling cascade is activated to switch on an oocyte phospholipase C leading to increased production of inositol 1,4,5-triphosphate (IP_3_) and diacylglycerol (DAG) [43]. DAG activates PKC and IP_3 _triggers Ca^2+ ^release from intracellular stores. The observation that oocytes exposed to the RGD peptide undergo a significant loss of cortical granules further suggests that under, our experimental conditions, a RGD-sensitive receptor on the oolemma has activated signalling pathways similar to those triggered by sperm at fertilization. Moreover, the occurrence of cortical granule exocytosis in association with an increased PKC activity supports the role for this kinase in the regulation of this event. On the other hand, being the loss of cortical granules a Ca^2+^-dependent event [44], present results might be an indirect evidence that oocytes exposed to the RGD peptide had undergone an increase of intracellular Ca^2+ ^as suggested by a previous study [45]. There is still the possibility that PKC activity in response to the RGD peptide represents that observed at fertilization in the absence of a Ca^2+ ^signal and probably supported by Ca^2+^-independent PKC isotypes [31]. In this respect, previous results based on the use of PKC agonists and antagonists, suggested that exocytosis can be triggered independently either by Ca^2+ ^rise and PKC [46,47]. Oocyte exposure to a RGD peptide seems to be responsible for a reorganization of actin network similar to that induced by sperm [48]. Moreover, as discussed by Tsaadon et al. [49], PKC may regulate the cytoskeletal dynamic underlying exocytosis enabling the process of vesicle fusion with plasma membrane.

A further observation associated with present results is that RGD-associated signalling leads to PKC activation and cortical granule exocytosis but is not able to stimulate meiosis resumption. This supports the hypothesis that, in contrast to PKC activation achieved by pharmacological agonists, the activation of a PKC signalling through a receptor-mediated mechanism, is not the sufficient trigger for the activation of the anaphase-promoting complex/cyclosome (APC/C) pathway leading to meiosis resumption [32,50]. In contrast to our results in the mouse, in bovine oocytes a release of meiotic arrest is observed after exposure to RGD peptides [25], it is likely that RGD-sensitive receptors might be capable of activating additional pathways.

## Conclusion

Although further investigation will be needed, our results suggest that, at fertilization, a sperm membrane protein containing a RGD sequence may interact with a RGD-sensitive receptor on the oolemma activating a cascade of signalling pathways involved in oocyte activation. Given that in the mouse and bovine sperm injection can induce an abnormal Ca^2+ ^response with developmental consequences [51,52], it can be speculated that a possible role of integrin-mediated pathways may be that to cooperate with those activated in the cytosol by other sperm molecules [4] in order to correctly orchestrate oocyte activation events. Although studies on animal models must be interpreted with caution, this hypothesis raises the need to better investigate the consequences of skipping gamete interaction at surface level in a number of assisted reproductive technologies.

## Competing interests

The author(s) declare that they have no competing interests.

## Authors' contributions

CT conceived of the study and experimental design, contributed to the acquisition of data and wrote the manuscript.

MCC have made substantial contribution to experimental design, acquisition of data and manuscript drafting.
